# Hospital wastewater as a significant source of microplastics in Northern Iran: Abundance, polymer composition, and environmental risk assessment

**DOI:** 10.1371/journal.pone.0358340

**Published:** 2026-10-12

**Authors:** Kosar Kouhi, Atefeh Jabari, Yalda Hashempour

**Affiliations:** 1 Department of Environmental Health Engineering, Student Research Committee, Mazandaran University of Medical Sciences, Sari, Iran; 2 Department of Environmental Health Engineering, School of Health, Mazandaran University of Medical Sciences, Sari, Iran; 3 Department of Environmental Health Engineering, School of Public Health, Health Sciences Research Center, Mazandaran University of Medical Sciences, Sari, Iran; National Research and Innovation Agency, INDONESIA

## Abstract

**Background:**

Microplastics (MPs) are ubiquitous contaminants increasingly detected in aquatic environments and wastewater systems, raising concerns regarding ecosystem and human health. Hospital wastewater has been identified as a source of MPs entering municipal systems; however, data on their occurrence, characteristics, and associated risks remain limited. This study aimed to characterize MP contamination in hospital wastewater and assess the associated environmental and health risks.

**Methods:**

Wastewater samples were collected from the influent and effluent of a hospital wastewater treatment plant in northern Iran. MPs were isolated using stainless-steel sieving followed by hydrogen peroxide (H_2_O_2_) digestion. Abundance, morphology, and size distribution were determined through microscopic examination, and chemical composition was analyzed using Scanning Electron Microscopy coupled with Energy Dispersive X-ray (SEM-EDX) and micro-Raman spectroscopy. The Pollution Load Index (PLI) and Hazard Index (HI) were calculated to evaluate contamination levels and associated risks.

**Results:**

The analysis showed mean MP concentrations of 21.13 ± 12.8 particles L^-1^ in the influent and 1.45 ± 0.89 particles L^-1^ in the effluent, reflecting a removal efficiency of 93.13%. Morphologically, fibrous particles predominated (92–96%), with black as the most common color and sizes below 250 µm being the most prevalent. Identified polymer compositions included polyamide (PA), polyvinyl chloride (PVC), polyethylene terephthalate (PET), and polyurethane (PU). Based on the calculated indices, the hospital wastewater reached a PLI of 1.86 and an HI of 1416.45, which corresponds to hazard level IV, **primarily attributed to the high hazard score of** PVC **(5001) due to its carcinogenic monomer and additive leaching.**

**Conclusions:**

Hospital wastewater is a significant source of MPs, particularly hazardous polymers such as PVC and PU. These findings highlight the need for targeted mitigation strategies, including advanced treatment processes and reduced use of toxic polymers in healthcare settings, to minimize MP discharge and protect environmental and public health

## 1 Introduction

Plastics, encompassing a wide range of synthetic and natural polymeric materials, have become essential in modern society due to their durability, versatility, chemical resistance, and low cost [1]. Their extensive use across healthcare, textiles, packaging, electronics, and other industries has led to a rapid increase in global plastic production [2], reaching approximately 400–460 million tonnes annually in recent years, with projections indicating a rise from 464 Mt in 2020 to about 884 Mt by 2050 under current consumption scenarios [3,4]. However, improper waste management and environmental persistence have resulted in widespread plastic pollution across terrestrial and aquatic ecosystems [5].

In the environment, plastics undergo physical, chemical, and biological degradation, fragmenting into microplastics (MPs), defined as particles smaller than 5 mm [6]. MPs include both primary forms, intentionally produced at microscopic sizes, and secondary forms derived from the breakdown of larger plastics [7].

Due to their small size, large surface area, and stability, MPs are now recognized as persistent environmental contaminants detected in water, sediments, soil, air, and biota [8]. Wastewater treatment plants (WWTPs) are major pathways for MP transfer from urban sources to aquatic environments. Although conventional treatment processes can remove a large fraction of MPs (48–99%), complete elimination is rarely achieved, resulting in continuous release into receiving waters and sludge systems [9]. Consequently, WWTPs remain significant secondary sources of MP pollution [10].

Hospital wastewater (HWW) represents a distinct, highly complex, and potentially high-risk effluent stream due to intensive water use and extensive reliance on plastic-based medical products. It contains a mixture of pharmaceuticals, disinfectants, surfactants, pathogens, and emerging micropollutants, making it environmentally relevant and potentially hazardous [11,12]. With daily effluent volumes of up to 250–570 m^3^ for medium-sized facilities [13,14], HWW receives MPs from facility-specific pathways such as laundering of synthetic medical textiles, cleaning processes, laboratory activities, kitchen operations, and disposal of plastic-based medical products [15,16]. Recent studies report high MP loads in hospital effluents, particularly in laundry wastewater, with concentrations reaching up to 2 × 10^5^ particles L^-1^, and in some cases up to 1.4 × 10^6^ particles L^-1^, predominantly composed of synthetic fibers such as PET and polyamides (PA), indicating hospitals as significant point sources of MP emissions into wastewater systems [17].

Beyond their physical presence, MPs have been suggested in previous studies to potentially act as a “Trojan horse” vector for antibiotic-resistant bacteria (ARB) and antibiotic resistance genes (ARGs), which may contribute to their persistence and dissemination in wastewater environments

[7,18]. They have also been reported to adsorb hydrophobic organic pollutants such as polycyclic aromatic hydrocarbons (PAHs), polychlorinated biphenyls (PCBs), and heavy metals, a behavior generally attributed to their surface area and physicochemical properties [19]. Toxicological studies further indicate that MPs may induce oxidative stress, inflammation, and reproductive toxicity, with effects depending on particle properties and exposure conditions [3].

Despite increasing attention to MPs in municipal wastewater systems, hospital wastewater treatment plants (HWWTPs) remain poorly investigated, particularly regarding MP abundance, polymer composition, morphological characteristics, and environmental risk profiles. Addressing these knowledge gaps is essential for understanding the role of hospital effluents as sources of MP pollution and for developing effective mitigation strategies.

In this context, the present study aims to investigated MP contamination in a HWWTP. Specifically, the study focused on (i) quantifying MP abundance, (ii) characterizing particle morphology (size, shape, and color), (iii) identifying polymer composition using micro-Raman spectroscopy, and (iv) evaluating environmental risk using established pollution and hazard indices. This integrated approach provides a comprehensive assessment of hospital-derived MP pollution and supports the development of targeted wastewater management strategies.

## 2 Materials and methods

### 2.1 Sampling sites

Wastewater samples were collected from the Imam Khomeini HWWTP in Sari, northern Iran. The hospital is located at coordinates 53° 3’ 2.892” N, 36° 33’ 55.340” E, at an altitude of 39 meters. It has 410 beds and 25 wards and provides a wide range of specialized healthcare services, including an emergency department, outpatient chemotherapy, endoscopy, dialysis, diagnostic imaging facilities (radiology, CT, and MRI), and pathology services. In addition, the hospital contains 11 operating rooms performing neurosurgical, gynecological, vascular, orthopedic, urological, and general surgical procedures. Wastewater from all hospital wards enters the HWWTP. All specialized wastewater streams, including those from laboratories, operating rooms, and dialysis units, are combined into a single collection network before entering the treatment plant. Therefore, no separate pre-treatment or segregation of these streams occurs at the source. The facility has a treatment capacity of 130 m^3^/day and employs an extended aeration activated sludge process to effectively remove organic matter and nutrients (such as biological nitrogen and phosphorus) as pre-treatment before discharging the treated effluent to a municipal wastewater treatment plant. The HWWTP encompasses various units, including screening, pre-aeration pond, aeration with sludge return system, sedimentation, and chlorination.

This study did not involve human participants, animals, or personal data. Sampling and analysis were conducted in accordance with institutional and national environmental research guidelines. Ethical approval was obtained from the Ethics Committee of Mazandaran University of Medical Sciences (Ethics Code: IR.MAZUMS.REC.1402.180; Tracking Code: 20685).

### 2.2 Sampling procedure

Samples were collected from two precise locations within the HWWTP: (i) the influent point immediately after the screening unit (before the pre-aeration tank), and (ii) the final effluent point after chlorination (just before discharge to the municipal wastewater system). Sampling was conducted in July and August 2023 using a time-composite strategy. In each sampling event, subsamples were collected every 2 hours over an 8-hour working shift and pooled into a single composite sample per location. A total of six sampling events were performed (three events per month).

The filtered volumes were 30 L per event (total 180 L) for influent and 270 L per event (total 1,620 L) for effluent. These volumes were filtered on-site through two removable stainless steel mesh screens with pore sizes of 5 mm and 25 µm [20]. The lower influent volume was chosen to prevent rapid clogging of the sieves due to the high loads of MPs and organic matter. In contrast, the substantially larger volume of treated effluent was necessary to compensate for the much lower MP concentration after the extended-aeration activated-sludge process, sedimentation, and chlorination, thereby ensuring a sufficient number of particles for reliable quantification, morphological characterization, and polymer identification.

The residues retained on the sieves were rinsed with 1000 mL of ultrapure water (pre-filtered through 0.45 µm membranes) and transferred into pre-cleaned dark glass bottles. Immediately after collection, the samples were placed in portable coolers with ice packs and transported to the laboratory at 4°C. Upon arrival, they were stored at 4°C in a dedicated refrigerator until further processing. All sample processing (digestion, density separation, and filtration) was completed within one week of collection to minimize any potential changes or contamination.

### 2.3 Laboratory analysis of samples

To extract MPs, samples were subjected to a pre-treatment process to remove all organic and inorganic matter. The protocol primarily involved oxidation for organic matter removal. Hydrogen peroxide (H_2_O_2_) was used as an oxidizing agent, which can digest organic matter without degrading polymer structures. Following this, density separation using a salt solution was applied to remove inorganic particles from the samples [20]. Samples were poured into a beaker, and 30% H_2_O_2_ was added until it constituted 5% of the total sample volume. The mixture was heated at 60 °C and stirred for one hour. After resting for a full day and night, they were kept in an oven at a temperature of 60 °C to ensure complete evaporation of H_2_O_2_ and water, thus drying the sample. A ZnCl_2_ solution with an approximate density of 1.6 g cm^−3^ was used due to its high recovery efficiency, cost-effectiveness, recyclability, and ability to separate inorganic particles such as sand. The ZnCl_2_ solution was mixed with the samples and allowed to settle for 24 hours. The supernatant, containing the potential MP fraction, was carefully collected and filtered onto glass fiber filters (S&S, Germany; 4–7 µm pore size). This separation procedure was repeated three times on the same filter to improve recovery efficiency [21].

### 2.4 Characterization of MPs

The physical characteristics of the MP samples were analyzed using an optical microscope (ZSM-1001, Iran) [22]. To measure particle size, an optical microscope was used in combination with a calibrated 250 µm stainless steel probe for accurate sizing. Image analysis was performed using ImageJ software, enabling quantitative assessment of particle size and shape. This combination of optical microscopy and digital image processing ensured reliable measurement and methodological transparency.

MPs were counted and categorized into four size classes: ≤ 250, 250–500, 500–1000, and 1000–5000 µm, and classified into four morphologies: fibers, spherules, sheets, and fragments [23]. Particle color was recorded as black, red, blue, white, yellow, and green.

The polymer types were identified using µ-Raman spectroscopy (Horiba, France) using 785 nm laser and a Raman shift range of 50–3500 cm^−1^. Raman spectroscopy is a non-destructive technique that generates detailed molecular spectra through the inelastic scattering of monochromatic laser light. It is known for its high spatial resolution and capability to detect features at submicron or nanometer scales, even in trace samples. Additionally, Raman spectral imaging enables the identification of molecular species within a sample and their visualization as two-dimensional chemical maps, without the need for prior labeling of MP particles [24].

The obtained spectra were matched against reference spectra of common polymers stored in spectral libraries for comparison and validation. Non-plastic particles were excluded based on spectral analysis. Of the 20 randomly selected particles, 18 were successfully identified using the Horiba Common Polymers Library.

In addition, Scanning Electron Microscopy coupled with Energy Dispersive X-ray spectroscopy (SEM-EDX; TESCAN Vega 3) was used for elemental analysis of selected particles to further confirm their composition [25]. MP abundance in influent and effluent samples was calculated as mean values from three replicates (particles L^-1^) [26]. Statistical analysis, including frequency, mean, and standard deviation, was performed using IBM SPSS Statistics 20.

### 2.5 Quality Assurance and Quality Control (QA/QC)

To prevent contamination during sampling and laboratory processing, all procedures were conducted in a controlled environment. Samples were handled within a laminar flow cabinet under low-airflow conditions, and direct contact with plastic materials was strictly avoided. Personnel wore cotton laboratory coats and face masks throughout all experimental steps to minimize airborne contamination.

All glassware, sieves, and forceps were thoroughly cleaned with ultrapure water and ethanol where applicable, and dried at 120°C prior to use. Chemical solutions were filtered before application, and all working surfaces were regularly cleaned and immediately covered with aluminum foil to prevent airborne deposition. Containers were sealed or covered when not in use to avoid external contamination. Quality control was assessed using procedural blanks, water blanks, and airborne contamination controls (open Petri dishes). Four water blanks and two Petri dishes were processed alongside the samples. Only one MP particle was detected in the water blanks (0.25 particles L^-1^), while no particles were found in the Petri dishes, indicating negligible contamination. Therefore, no blank correction was applied to the reported concentrations.

Regarding method performance, recovery efficiency tests using spiked MP particles were not conducted in this study, which represents a methodological limitation. However, previous studies have demonstrated that the combination of H_2_O_2_ digestion and ZnCl_2_ density separation provides high extraction efficiency across a wide range of polymer types, supporting the reliability of the applied method MP [27].

### 2.6 Risk assessment

Risk assessment was performed using the Polymer Hazard Index (HI), which considers the toxicity of different polymer types, and the Pollution Load Index (PLI), which reflects MP abundance.

The HI was calculated as follows:


$$\begin{array}{c} HI=\sum Pn\times Sn, \end{array}$$ (1)

where HI is the calculated polymer risk index; Pn is the percentage of each MP polymer type at each sampling site; and Sn is the score for the polymers comprising the MPs [28,29].

The PLI was calculated using the following equations:


$$\begin{array}{c} CFi=\frac{Ci}{C\text{0}i}, \end{array}$$ (2)

$$\begin{array}{c} PLI=\sqrt{CFi}, \end{array}$$ (3)

where Ci represents the MP concentration in each sample, Coi is the minimum MP concentration observed in the study, taken as the reference value, and CFi is the concentration factor calculated for each sample [30]. The risk scores and their classification are presented in Table 1 and Table 2, respectively.

**Table 1:** Hazard scores of selected MPs polymer type [28,29].

Polymer	Abbreviation	Score
Polyvinyl Chloride	PVC	5001
Thermoplastic Polyurethanes	TPU	1094
Polyamide (Nylon)	PA	47
Polyethylene Terephthalate	PET	4

**Table 2:** Risk level categories for MP pollution according to the UN Globally Harmonized System [30].

	Risk level categories			
Index	I	II	III	IV
HI	< 10	10–100	100-1000	> 1000
PLI	< 10	10-20	20-30	> 30

## 3 Results

### 3.1 Abundance of the MP particles and removal efficiency

The abundance of MPs in the HWW samples was 21.13 ± 12.8 particles L^-1^ in the influent and 1.45 ± 0.89 particles L^−1^ in the effluent, corresponding to an overall removal efficiency of 93.13% achieved by the treatment processes. Monthly analysis indicated that the mean influent MP concentration was higher in July (month 1; 25.8 ± 16.36 particles L^-1^) than in August (month 2; 16.46 ± 9.05 particles L^-1^). The corresponding effluent MP concentrations were 0.81 ± 0.35 and 2.08 ± 1.01 particles L^-1^, yielding removal efficiencies of 96.86% and 87.36%, respectively. Influent MP concentration ranged from a minimum of 9.9 particles L^-1^ to a maximum of 43.6 particles L^-1^, with associated removal efficiencies of 95.75% and 92.64%.

### 3.2 The physical characteristics of MPs in the HWWTP

The physical analysis of isolated MPs revealed distinct characteristics regarding their color, shape, and size. Black was the predominant color in both influent and effluent samples, accounting for 48% and 41% of the identified particles, respectively. Blue particles represented 22% of the influent and 21% in effluent samples (Fig 1a).

**Fig 1:**
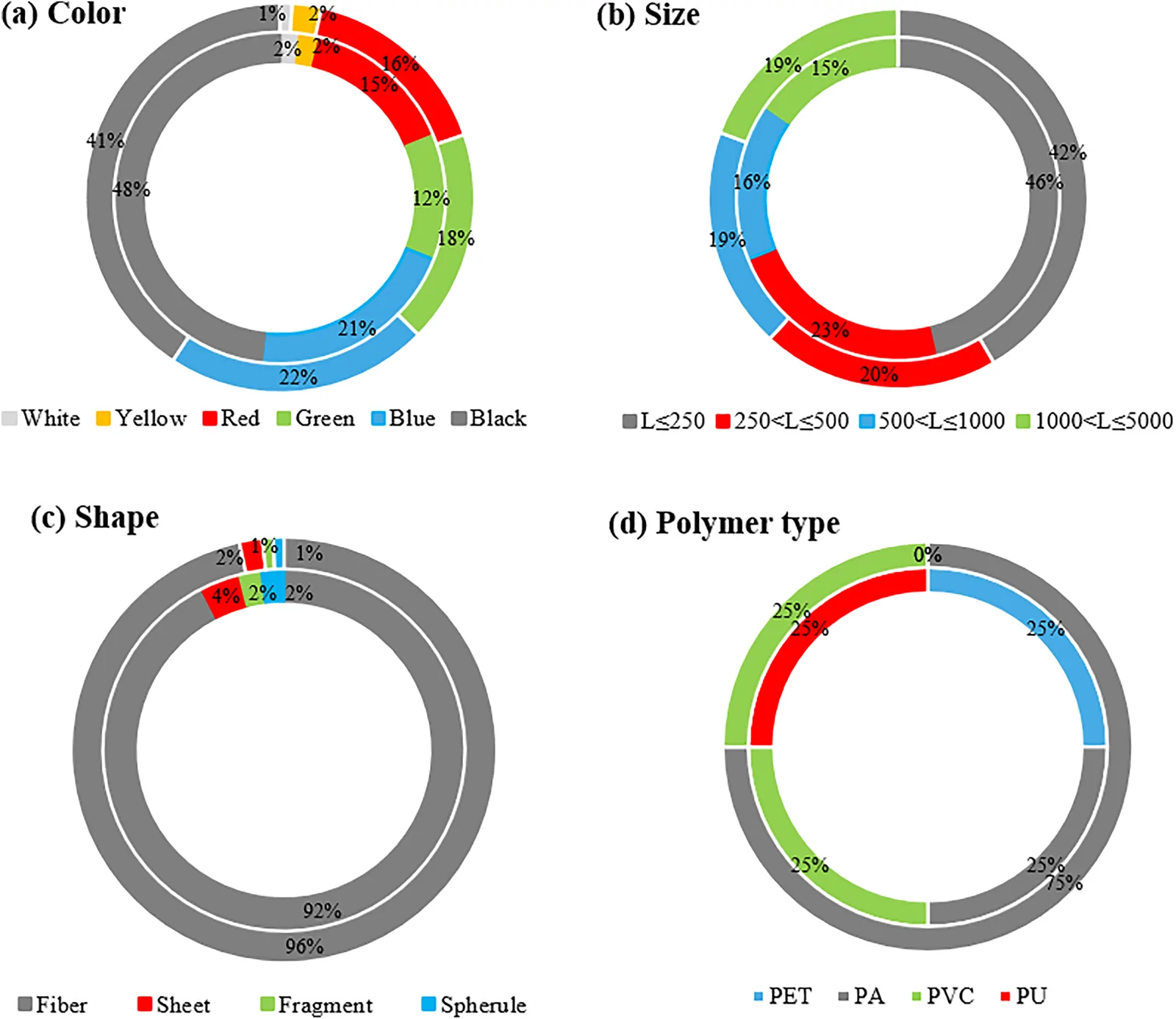
Comparison of the (a) Color, (b) Size, (c) Shape, and (d) Polymer of MPs in HWW (In diagram a-c, outer circle = effluent and inner circle = influent).

In terms of morphology, fibers were identified as the main form of MPs, constituting 92–96% of all samples (Fig 1c).

Regarding particle size, the dominant size range for MP particles in influent and effluent samples was ≤ 250 μm, accounting for 46% and 42% of total MP particles, respectively (Fig 1b). Overall, 69% of the MP particles in influent and 62% in effluent samples were ≤500 μm.

Overall, black-colored particles, fibrous morphologies, and small-sized MPs dominated both influent and effluent samples.

### 3.3 MP screening by SEM/EDS

Scanning Electron Microscopy coupled with Energy Dispersive X-ray Spectroscopy (SEM/EDS) was used to analyze the surface morphology and elemental composition of MP particles. SEM images (Fig 2) illustrate representative particles identified from hospital influent and effluent. The plastic nature and polymer types of these MPs were confirmed by micro-Raman spectroscopy.

**Fig 2:**
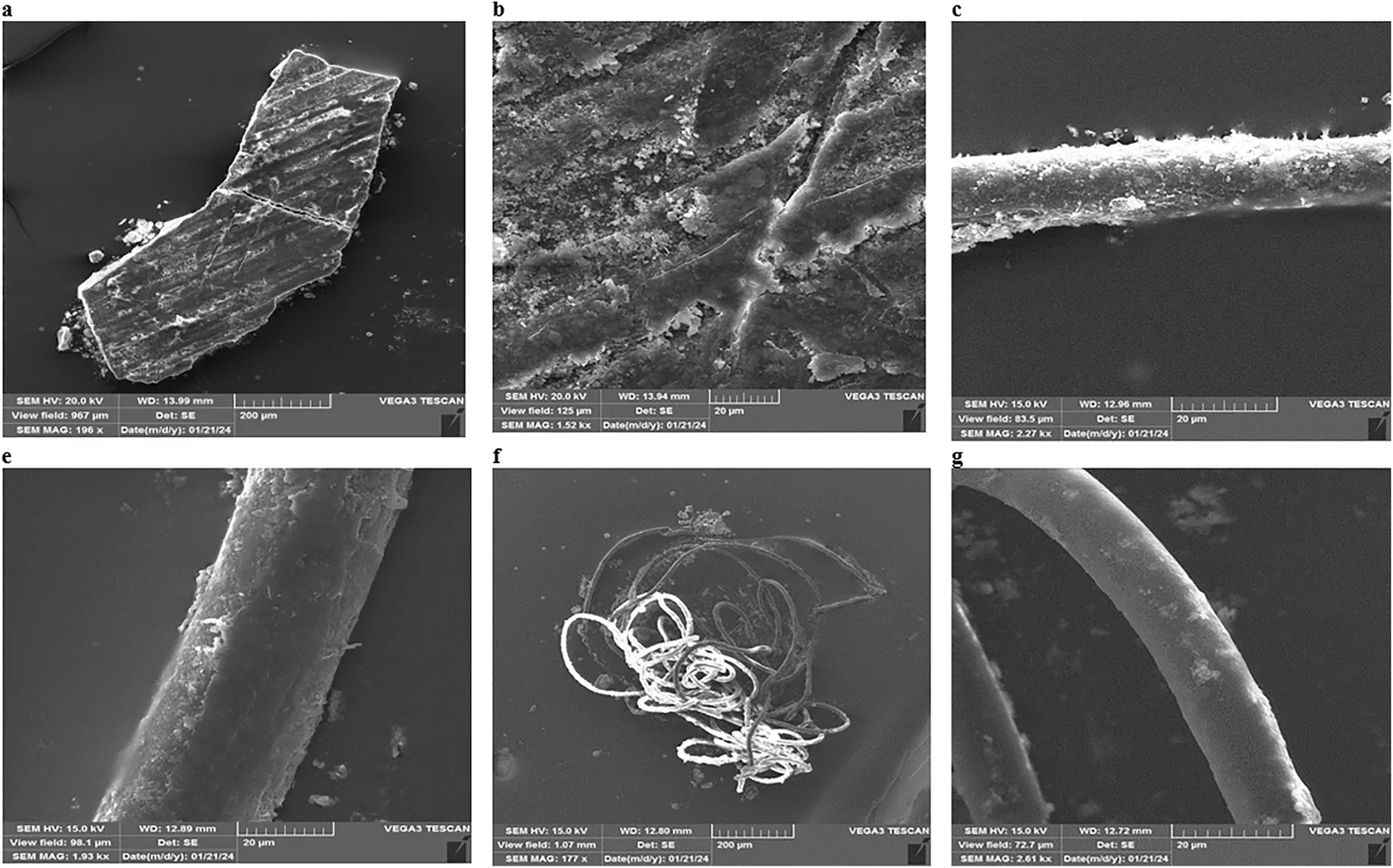
SEM Images of the Identified MP Particles from HWWTP.

SEM analysis was performed on six selected MPs representing four distinct polymer classes (PET, PU, PA, and PVC). Fig 2a shows red-colored fragments, while higher magnification (Fig 2b) reveals surface cracks consistent with oxidative weathering. Light microscope images of fibers (Fig 2c, 2d, 2e, 2f) display blue and green colors, with higher-magnification SEM images (Fig 2d, 2f) revealing smooth surfaces and uniform cross-sections.

Features including cracks, flakes, pores, adhering particles, and rough textures were observed in several particles. The elemental composition of the analyzed MPs, presented in Table 3, provides further insight into the polymer types previously identified by µ-Raman spectroscopy. In particular, the detection of chlorine (Cl) in particles I and II (2.14% and 1.73%, respectively) strongly supports the assignment of these particles to PVC, as chlorine is a characteristic constituent of PVC structure. In contrast, the presence of elements such as Zn, Ba, and Ti is not related to the polymer backbone itself but is more likely associated with polymer additives (e.g., stabilizers, fillers) or residual ZnCl_2_ from density separation, indicating chemical complexity within the identified polymers (PET, PU, PA, and PVC)

**Table 3:** EDS Report of Identified MP Particles.

Particle	C	O	Na	Cl	Zn	Al	Si	N	S	Ti	Ba	Mo
I	50.57	24.54	1.45	2.14	4.52	0.22	0.25	–	3.07	2.27	9.69	1.28
II	62.68	26.33	4.41	1.73	4.86	–	–	–	–	–	–	–
III	59.12	39.18	0.30	0.71	0.7	–	–	–	–	–	–	–
IV	34.03	26.09	9.39	6.35	6.43	0.70	–	17.02	–	–	–	–

### 3.4 MP identification by µ-Ramans spectroscopy

Determining the chemical composition of MPs is essential for identifying the types of polymers and inferring their potential sources. In this study, µ-Raman spectroscopy was employed as a high-resolution, non-destructive analytical technique to characterize polymer composition based on molecular vibrational signatures. Fig 3 presents representative µ-Raman spectra used for polymer identification of MPs in HWW samples.

**Fig 3:**
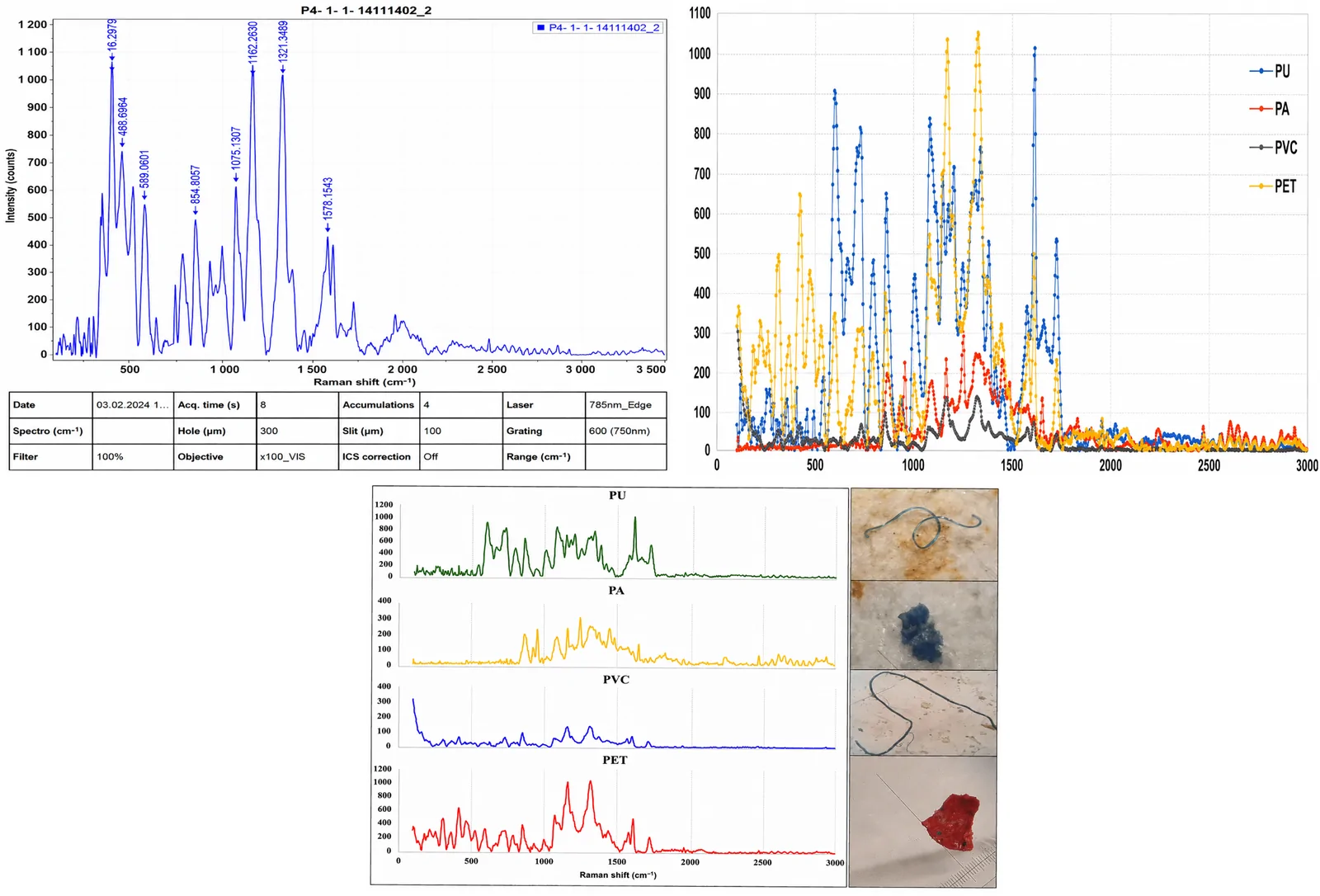
Examples of µ-Raman spectroscopy peaks for identifying the polymer type of MPs in HWW samples.

Raman mapping further enabled spatially resolved identification of MPs, as spectra collected across fixed acquisition regions varied depending on the step size. At each point, a full spectral range (50–3400 cm^-1^) was obtained and compared against reference libraries for polymer identification. The use of automated spectral matching software significantly improved identification accuracy and reproducibility [31].

A total of four polymer types were identified across all HWW samples (Fig 1d). PA and PVC were the dominant polymers, followed by PU and PET.

### 3.5 Risk assessment

The calculated HI and PLI for HWW were 1416.45 and 1.86, respectively. As shown in Table 1, PVC possesses the highest hazard score (5001), which largely drives the elevated HI value due to its dominance in the polymer composition. According to the risk classification presented in Table 2, the HI classifies MP pollution as hazard level IV (>1000), while the PLI falls within risk level I (<10). The PLI was calculated using the minimum MP concentration observed in this study (9.9 particles L^-1^) as the reference baseline (C₀). Therefore, the PLI reflects the relative MP abundance in the studied HWW with respect to the lowest concentration measured during the sampling period. The low PLI indicates a relatively low pollution level based on MP abundance, whereas the elevated HI is primarily driven by the predominance of the high-hazard polymer PVC.

## 4 Discussion

### 4.1 Performance of the HWWTP in MP removal

The present study demonstrated that the extended aeration activated sludge system achieved a high MP removal efficiency of 93.13%, indicating that this treatment process is effective in reducing MP concentrations in hospital wastewater. The observed differences between the two sampling periods may be associated with variations in wastewater composition arising from changing inflow patterns (e.g., increased hospital activity or seasonal shifts in product usage) as well as operational conditions of the treatment plant. Consistent with the present findings, Kamani et al. (2024) reported MP concentrations of 140 particles L^-1^ in influent and 15 particles L^-1^ in effluent HWW, corresponding to a removal efficiency of 98.28%. Although influent concentrations were substantially higher than those observed in the present study, both investigations demonstrated the capability of biological treatment systems to achieve considerable MP removal in HWW environments [32]. Additional evidence from other wastewater treatment studies further underscores the importance of treatment configuration. Maw et al. (2024), for example, reported that a two-stage activated sludge process achieved a removal efficiency of 96.8%, highlighting the role of specific operational variables such as Hydraulic Retention Times (HRTs) and flow dynamics in enhancing MP removal [33]. Similarly, Zhang et al. (2023) demonstrated that variations in activated sludge design can significantly influence MP removal performance [34].

In contrast, lower removal efficiencies have also been documented. Liu et al. (2019) reported only 16.6% MP removal in a wastewater treatment plant relying primarily on primary sedimentation with limited biological treatment. These differences indicate that MP removal is strongly governed by treatment configuration and physical separation processes‒ particularly particle aggregation, flocculation, settling, and sludge retention during the activated sludge and sedimentation stages‒ while disinfection provides limited additional removal of MPs [35]. The variability in reported MP concentrations among HWW studies can be attributed to differences in hospital size, healthcare activities, patient load, medical services provided, wastewater management practices, and other local factors affecting MP inputs [36,37].

Moreover, the presence of MPs in human excreta‒ with reported concentrations of 2589 ± 2931 particles L^-1^ in urine [38] and 41.8 particles L^-1^ in feces [39] ‒ underscores the human contribution to MP loads in wastewater systems. Consumer products, including cleaning agents and medical supplies, are also recognized as significant sources of MPs entering WWTPs [40–42]. Collectively, these findings emphasize the importance of implementing source-control strategies to reduce MP emissions at the origin.

### 4.2 Possible sources and characteristics of MPs in HWW

The predominance of black-colored particles, fibrous MPs, and smaller size fractions observed in the present study is consistent with previous reports on HWW systems. Kamani et al. (2024) reported that black-colored particles and fibrous MPs were the dominant categories in hospital effluents, with most particles occurring within the smaller size fractions. The high abundance of fibers in both studies is likely linked to the extensive use and laundering of synthetic medical textiles in healthcare facilities [32]. Similar patterns have been documented elsewhere. Uogintė et al. (2022) found that MP collected with a 20–1000 μm mesh size predominantly exhibited black, white, and clear colors [43]. Hajji et al. (2023) likewise reported that red, black, blue, and transparent particles were the most prevalent colors [44]. In the present study, black and blue MPs dominated both influent and effluent samples, while red, green, yellow, and white particles were less frequent. This distribution may reflect regional variations in MP color profiles driven by local environmental conditions and antropogenic activities [44]. Additionally, aging, leaching, or UV-induced oxidation of brightly colored particles can contribute to the predominance of black or transparent MPs [45].

Morphological finding further align with the literature. Alavian Petroudi et al. (2021) reported that MPs in the Sari city wastewater treatment plant were mainly fiber-shaped, and ranged from 37 to 300 μm in size [46]. Arregocés-Garcés et al. (2024) found fibers to constitute 67–75% of MP in wastewater [47], while Bayo et al. (2020) observed that fragments and fibers in the 400–600 µm range were more abundant in effluent samples [36]. The consistent dominance of fibrous MPs across studies underscores their persistence in wastewater environments. The elevated proportion of fibers in effluent samples is of particular concern because of their abundance and poteitial ecological risks [48].

Fragments, by contrast, may originate from the degradation of plastic materials used in hospital settings [49].

The predominance of fibrous MPs is most likely associated with the release of synthetic fibers during the washing of medical textiles, a recognized major pathway for MP entry into aquatic systems [50].These results are consistent with findings by Franco et al. [51] and Sakali et al. [52], which indicate that smaller MPs tend to remain suspended in the aqueous phase and are therefore less efficiently removed during environmental,treatment processes [22].

Previous studies have also suggested that smaller-sized MPs may reduce disinfection efficiency by providing protective niches for microorganisms, potentially allowing them to evade treatment [45]. Consequently, the high proportion of small MPs (≤500 μm) represents a significant environmental and public health concern.

Overall, the observed predominance of black and blue particles, the prevalence of smaller-sized MPs (≤500 μm), and the high abundance of fibers highlight the need for further investigating into the sources, transport pathways, and environmental impacts of MPs in HWW systems. A deeper understanding of these characteristics is essential for developing effective mitigation and management strategies.

### 4.3 Surface and chemical characterization of MPs

SEM analysis revealed that the examined MPs exhibited varying degrees of environmental weathering. While some particles displayed relatively smooth surfaces, others showed surface irregularities including cracks, burrs, cavities, flakes, adhering particles, pores, and rough textures. These morphological features are consistent with previous descripations of weathered MPs in aquatic environments [23,53]. The presence of cracks, cavities, and rough surfaces increase the specific surface area of MPs, thereby enhancing their potential to interact with surrounding contaminants and microorganisms [54,55]. Such surface modifications may influence the environmental behavior of MPs by increasing their capacity to adsorb pollutants or support microbial colonization.

EDS analysis complemented the µ-Raman results by providing additional insight into the elemental composition of the particles. The detection of chlorine (Cl) in particles I and II supports their identification as PVC, as chlorine is a characteristic constituent of the PVC polymer structure. In contrast, elements such as Zn, Ba, and Ti are more likely associated with polymer additives (e.g., stabilizers and fillers) or residual ZnCl_2_ from the density separation procedure rather than the polymer backbone itself. These findings underscore the chemical complexity of the identified MPs and are consistent with previous reports documenting additive-derived elements in environmental microplastics [23].

### 4.4 Polymer composition in comparison with previous studies

The predominance of PA and PVC observed in the present study points to a substantial contribution from textile- and medical-related sources within hospital wastewater systems. This interpretation is supported by previous HWW investigations in which PA and PVC were identified as predominant polymers associated with medical textiles, tubing systems, and disposable healthcare products [32]. Comparable polymer profiles have been reported in other wastewater environments. Liu et al. (2019), for example, observed a higher proportion of PA relative to polyolefins in WWTPs [35], while Van Do et al. (2022) identified PET, nylon, and PVC as prevalent polymers in treated effluents [55]. Similarly, Asadi et al. (2023) and Xu et al. (2023) reported diverse polymer compositions including PA, PVC, PP, and rayon [37,56].

Notably, PU, a polymer commonly used in the manufacture of medical devices and implants, was also detected among the MPs in the wastewater from the studied hospital [57]. In the medical sector, more than 25% of polymer products‒ including flexible blood containers, intravenous solution bags, flexible tubes, and oxygen masks, are composed of PVC [58,59]. Personal protective equipment such as masks, safety goggles, face shields, and hair covers is frequently manufactured from plastics such as PET [60], while PA is didely employed in medical textiles [61,62].

PVC is classified among the more hazardous polymers and ranks as the third most produced plastic globally, with an annual production of approximately 37 million tonnes. Owing to its carcinogenic monomer and the substantial quantities of potentially hazardous additives required in its formulation—many of which are prone to leaching—PVC warrants particular attention in environmental and public health assessments [28].

Overall, the polymer composition identified in this study reflects the specific operational characteristics of hospital environments and underscores the need for targeted strategies to minimize the release of polymer-based MPs into wastewater systems.

### 4.5 Environmental implications of the observed risk levels

The contrasting HI and PLI values obtained in the present study demonstrates that the environmental risk posed by MPs cannot be evaluated solely on the basis of particle abundance; polymer composition is equally critical in hazard assessment. Although the calculated PLI indicated a relatively low pollution level, the predominance of PVC‒ the polymer with the highest hazard score among thos identified‒ resulted in an elevated HI value. A comparable association between PVC dominance and high HI values has been reported by Zhou et al. [63] and Ren et al. [29], reinforcing the importance of polymer identity in determining overall hazard classification.

While HI and PLI provide useful frameworks for categorizing MP pollution, their interpretation shoulrequires caution because of inherent methodological limitations. Current risk models do not fully capture the ecological risk of MPs, and standardized assessment protocols remain lacking. Variations in sampling techniques can substantially influence measured MP abundances and, consequently, the outcomes of HI calculations. Dusacuy et al. [64] notedhted that different collection methods yield diverse results, with one comparative study showing that trawl-collected MPs were significantly lower in abundance than those recovered by other filtration approaches [65]. Furthermore, the HI primarily evaluates polymer hazards on the basis of monomer composition and does not adequately account for the potential contribution of additives‒whwther intentionally incorporated during manufacturing or subsequently adsorbed from the surrounding environment. As a result, the overall toxicity of MPs may be driven more by the complex mixture of associated chemicals than by the polymer particles themeselves [66].

These considerations underscore the necessity of proper treatment and management of high-risk polymers, particularly PVC, prior to environmental discharge in order to mitigate potential ecological and human health impacts.

### 4.6 Control measures for MPs

The extensive global production of plastic presents significant management challenges. The most effective strategy to mitigate MP pollution is through source control. In the short term, society must limit the unnecessary application of non-reusable plastic products, such as bottled water, plastic bags, drinking straws, and disposable utensils. Additionally, the government must prioritize the enhancement of waste collection and recycling initiatives to minimize environmental waste [67]. Control strategies for MPs can be systematically categorized into three hierarchical levels: production, application, and discharge stages. The continuous increase in plastic production and consumption is directly associated with higher volumes of plastic waste and subsequent MP generation. Therefore, mitigation efforts should target all stages of the plastic life cycle to effectively reduce MP release into the environment [68]. In the context of the present study, the observed dominance of fibrous MPs together with PA and PVC polymers suggests that hospital textiles and single-use medical plastic products represent key local sources of contamination. To effectively manage source control, Lv et al. (2019) [69] recommend several measures, including the restriction or prohibition of primary MPs (e.g., microbeads), polystyrene-based materials (e.g., Styrofoam), and lightweight plastic bags, as well as reducing microfiber release from textile laundering processes. Given the high abundance of fibers identified in this study, mitigation strategies targeting hospital laundry systems and medical textile management may be particularly relevant. Furthermore, technological improvements in WWTPs can enhance the removal efficiency of MPs at the discharge stage. Although the studied treatment system achieved high overall removal efficiency, the persistence of MPs in the effluent indicates that advanced or tertiary treatment processes may further reduce environmental release.

Effective management of MP pollution requires a coordinated and multi-stakeholder approach, involving both governmental authorities and industrial sectors. This includes the implementation of stringent regulations, development of supportive policies, and promotion of sustainable production and consumption patterns [69]. Such integrated strategies are critical for achieving long-term reduction of MP contamination in environmental systems.

## 5 Conclusion

This study provides important insights into MP pollution in HWW. The treatment plant achieved 93.13% MP removal, but smaller MPs (≤250 µm) and fibrous particles (92–96%) persisted in the effluent, raising concerns about their release into the environment. Polymer analysis revealed hazardous types, notably PVC (hazard score 5001), which drove the Hazard Index to level IV (HI = 1416.45). In contrast, the Pollution Load Index remained low (PLI = 1.86, level I), indicating that MP abundance alone does not reflect toxicity.

These findings confirm that HWW is a significant point source of toxic MPs, especially PVC and PU, which are widely used in medical products. To mitigate environmental and health risks, we recommend: (i) replacing hazardous polymers in healthcare settings where feasible, (ii) upgrading treatment technologies to capture smaller MPs, and (iii) implementing routine monitoring with standardized protocols. Future research should include spiked recovery tests and assess additive leaching from MPs. Policymakers should consider hospital-specific regulations to limit MP discharge into municipal systems.

## References

[1] Franco AA, Martín-García AP, Egea-Corbacho A, Arellano JM, Albendín G, Rodríguez-Barroso R, et al. Assessment and accumulation of microplastics in sewage sludge at wastewater treatment plants located in Cádiz, Spain. Environ Pollut. 2023;317:120689. doi: 10.1016/j.envpol.2022.120689 36435286

[2] Jabbari A, Zazouli M-A, Hashempour Y. Investigating the presence of microplastics as an emerging contaminant in water and various beverages: a review study. Journal of health research in community. 2024. https://dor.isc.ac/dor/20.1001.1.24236772.1403.10.2.8.7

[3] Adib D, Mafigholami R, Tabeshkia H. Identification of microplastics in conventional drinking water treatment plants in Tehran, Iran. J Environ Health Sci Eng. 2021;19(2):1817–26. doi: 10.1007/s40201-021-00737-3 34900309 PMC8617154

[4] Dokl M, Copot A, Krajnc D, Fan YV, Vujanović A, Aviso KB, et al. Global projections of plastic use, end-of-life fate and potential changes in consumption, reduction, recycling and replacement with bioplastics to 2050. Sustainable Production and Consumption. 2024;51:498–518. doi: 10.1016/j.spc.2024.09.025

[5] Zazouli M, Nejati H, Hashempour Y, Dehbandi R, Nam VT, Fakhri Y. Occurrence of microplastics (MPs) in the gastrointestinal tract of fishes: a global systematic review and meta-analysis and meta-regression. Sci Total Environ. 2022;815:152743. doi: 10.1016/j.scitotenv.2021.152743 35007572

[6] Yaseen A, Assad I, Sofi MS, Hashmi MZ, Bhat SU. A global review of microplastics in wastewater treatment plants: understanding their occurrence, fate and impact. Environ Res. 2022;212(Pt B):113258. doi: 10.1016/j.envres.2022.113258 35430276

[7] Tuvo B, Scarpaci M, Bracaloni S, Esposito E, Costa AL, Ioppolo M, et al. Microplastics and antibiotic resistance: the magnitude of the problem and the emerging role of hospital wastewater. Int J Environ Res Public Health. 2023;20(10):5868. doi: 10.3390/ijerph20105868 37239594 PMC10218389

[8] Zhang F, Wang Z, Vijver MG, Peijnenburg WJGM. Theoretical investigation on the interactions of microplastics with a SARS-CoV-2 RNA fragment and their potential impacts on viral transport and exposure. Sci Total Environ. 2022;842:156812. doi: 10.1016/j.scitotenv.2022.156812 35738381 PMC9212631

[9] Kieu-Le T-C, Ngo T-P, Lai M-T, Pham N-H, Nguyen N-B-T, Nguyen P-D, et al. Microplastics in wastewater and sludge from centralized industrial wastewater treatment plants in Ho Chi Minh City, Vietnam. Case Studies in Chemical and Environmental Engineering. 2025;12:101276. doi: 10.1016/j.cscee.2025.101276

[10] Hashempour Y, Jabari A, Kouhi K, Fendereski A. Introducing statistical methods to identify the sources of microplastics in the aquatic environment: an overview. Journal of health research in community. 2024;9(4):111–20. https://dor.isc.ac/dor/20.1001.1.24236772.1402.9.4.10.6

[11] Khan AH, Khan NA, Ahmed S, Dhingra A, Singh CP, Khan SU, et al. Application of advanced oxidation processes followed by different treatment technologies for hospital wastewater treatment. Journal of Cleaner Production. 2020;269:122411. doi: 10.1016/j.jclepro.2020.122411

[12] Magdaleno A, Juárez AB, Dragani V, Saenz ME, Paz M, Moretton J. Ecotoxicological and genotoxic evaluation of Buenos Aires city (Argentina) hospital wastewater. J Toxicol. 2014;2014:248461. doi: 10.1155/2014/248461 25214834 PMC4158310

[13] Gan S, Wu P, Song Y, Guo P, Cai N, Yuan F, et al. Non-targeted characterization of dissolved organic matter from a wastewater treatment plant by FT-ICR-MS: a case study of hospital sewage. Journal of Water Process Engineering. 2022;48:102834. doi: 10.1016/j.jwpe.2022.102834

[14] Oliveira TS, Al Aukidy M, Verlicchi P. Occurrence of common pollutants and pharmaceuticals in hospital effluents. Hospital wastewaters: characteristics, management, treatment and environmental risks. 2018. p. 17–32. doi: 10.1007/698_2017_9

[15] Wang C, Chen W, Zhao H, Tang J, Li G, Zhou Q, et al. Microplastic fiber release by laundry: a comparative study of hand-washing and machine-washing. ACS EST Water. 2023;3(1):147–55. doi: 10.1021/acsestwater.2c00462

[16] Gopinath PM, Parvathi VD, Yoghalakshmi N, Kumar SM, Athulya PA, Mukherjee A, et al. Plastic particles in medicine: a systematic review of exposure and effects to human health. Chemosphere. 2022;303(Pt 3):135227. doi: 10.1016/j.chemosphere.2022.135227 35671817

[17] Bortot Coelho FE, Sohn SI, Candelario VM, Hartmann NIB, Hélix-Nielsen C, Zhang W. Microplastics removal from a hospital laundry wastewater combining ceramic membranes and a photocatalytic membrane reactor: fouling mitigation, water reuse, and cost estimation. Journal of Membrane Science. 2025;715:123485. doi: 10.1016/j.memsci.2024.123485

[18] Zhang M, Xu L. Transport of micro- and nanoplastics in the environment: Trojan-Horse effect for organic contaminants. Critical Reviews in Environmental Science and Technology. 2020;52(5):810–46. doi: 10.1080/10643389.2020.1845531

[19] Brennecke D, Duarte B, Paiva F, Caçador I, Canning-Clode J. Microplastics as vector for heavy metal contamination from the marine environment. Estuarine, Coastal and Shelf Science. 2016;178:189–95. doi: 10.1016/j.ecss.2015.12.003

[20] Dronjak L, Exposito N, Rovira J, Florencio K, Emiliano P, Corzo B, et al. Screening of microplastics in water and sludge lines of a drinking water treatment plant in Catalonia, Spain. Water Res. 2022;225:119185. doi: 10.1016/j.watres.2022.119185 36209664

[21] Magni S, Binelli A, Pittura L, Avio CG, Della Torre C, Parenti CC, et al. The fate of microplastics in an Italian Wastewater Treatment Plant. Sci Total Environ. 2019;652:602–10. doi: 10.1016/j.scitotenv.2018.10.269 30368189

[22] Xu X, Jian Y, Xue Y, Hou Q, Wang L. Microplastics in the wastewater treatment plants (WWTPs): occurrence and removal. Chemosphere. 2019;235:1089–96. doi: 10.1016/j.chemosphere.2019.06.197 31561299

[23] Mehdinia A, Dehbandi R, Hamzehpour A, Rahnama R. Identification of microplastics in the sediments of southern coasts of the Caspian Sea, north of Iran. Environ Pollut. 2020;258:113738. doi: 10.1016/j.envpol.2019.113738 31838395

[24] Shi Y, Shi L, Huang H, Ye K, Yang L, Wang Z, et al. Analysis of aged microplastics: a review. Environ Chem Lett. 2024;22(4):1861–88. doi: 10.1007/s10311-024-01731-5

[25] Sadeghian HA, Mirhosseini SA. The Correlation of Green School indicators with Life Quality of life of Female Students in First Level of High Schools in Yazd City in 2021-2020. Tolooebehdasht. 2023;21(5):80–94. doi: 10.18502/tbj.v21i5.11755

[26] Yuan F, Zhao H, Sun H, Sun Y, Zhao J, Xia T. Investigation of microplastics in sludge from five wastewater treatment plants in Nanjing, China. J Environ Manage. 2022;301:113793. doi: 10.1016/j.jenvman.2021.113793 34601347

[27] Rodrigues MO, Gonçalves AMM, Gonçalves FJM, Abrantes N. Improving cost-efficiency for MPs density separation by zinc chloride reuse. MethodsX. 2020;7:100785. doi: 10.1016/j.mex.2020.100785 32025505 PMC6997619

[28] Lithner D, Larsson A, Dave G. Environmental and health hazard ranking and assessment of plastic polymers based on chemical composition. Sci Total Environ. 2011;409(18):3309–24. doi: 10.1016/j.scitotenv.2011.04.038 21663944

[29] Ren X, Sun Y, Wang Z, Barceló D, Wang Q, Zhang Z, et al. Abundance and characteristics of microplastic in sewage sludge: a case study of Yangling, Shaanxi province, China. Case Studies in Chemical and Environmental Engineering. 2020;2:100050. doi: 10.1016/j.cscee.2020.100050

[30] Picó Y, Soursou V, Alfarhan AH, El-Sheikh MA, Barceló D. First evidence of microplastics occurrence in mixed surface and treated wastewater from two major Saudi Arabian cities and assessment of their ecological risk. J Hazard Mater. 2021;416:125747. doi: 10.1016/j.jhazmat.2021.125747 33819645

[31] Ling X, Cheng J, Yao W, Qian H, Ding D, Yu Z, et al. Identification and visualization of polystyrene microplastics/nanoplastics in flavored Yogurt by Raman Imaging. Toxics. 2024;12(5):330. doi: 10.3390/toxics12050330 38787108 PMC11125995

[32] Kamani H, Ghayebzadeh M, Azari A, Ganji F. Characteristics of microplastics in a hospital wastewater treatment plant effluent and hazard risk assessment. Environ Process. 2024;11(1):15. doi: 10.1007/s40710-024-00694-7

[33] Maw MM, Boontanon N, Aung HKZZ, Jindal R, Fujii S, Visvanathan C, et al. Microplastics in wastewater and sludge from centralized and decentralized wastewater treatment plants: effects of treatment systems and microplastic characteristics. Chemosphere. 2024; 2024:142536. doi: 10.1016/j.chemosphere.2024.142536 38844106

[34] Zhang B, Wu Q, Gao S, Ruan Y, Qi G, Guo K, et al. Distribution and removal mechanism of microplastics in urban wastewater plants systems via different processes. Environ Pollut. 2023;320:121076. doi: 10.1016/j.envpol.2023.121076 36641065

[35] Liu X, Yuan W, Di M, Li Z, Wang J. Transfer and fate of microplastics during the conventional activated sludge process in one wastewater treatment plant of China. Chemical Engineering Journal. 2019;362:176–82. doi: 10.1016/j.cej.2019.01.033

[36] Bayo J, Olmos S, López-Castellanos J. Microplastics in an urban wastewater treatment plant: the influence of physicochemical parameters and environmental factors. Chemosphere. 2020;238:124593. doi: 10.1016/j.chemosphere.2019.124593 31446275

[37] Xu N, Jiang L, Zhang Y, Shen Y, Wang Y, Wang S, et al. Microplastic pollution in the offshore sea, rivers and wastewater treatment plants in Jiangsu coastal area in China. Mar Environ Res. 2023;188:105992. doi: 10.1016/j.marenvres.2023.105992 37094527

[38] Rotchell JM, Austin C, Chapman E, Atherall CA, Liddle CR, Dunstan TS, et al. Microplastics in human urine: characterisation using μFTIR and sampling challenges using healthy donors and endometriosis participants. Ecotoxicol Environ Saf. 2024;274:116208. doi: 10.1016/j.ecoenv.2024.116208 38489903

[39] Yan Z, Liu Y, Zhang T, Zhang F, Ren H, Zhang Y. Analysis of microplastics in human feces reveals a correlation between fecal microplastics and inflammatory bowel disease status. Environ Sci Technol. 2022;56(1):414–21. doi: 10.1021/acs.est.1c03924 34935363

[40] Lin Q, Pang L, Ngo HH, Guo W, Zhao S, Liu L, et al. Occurrence of microplastics in three types of household cleaning products and their estimated emissions into the aquatic environment. Sci Total Environ. 2023;902:165903. doi: 10.1016/j.scitotenv.2023.165903 37524188

[41] Çağlayan U, Gündoğdu S, Ramos TM, Syberg K. Intravenous hypertonic fluids as a source of human microplastic exposure. Environ Toxicol Pharmacol. 2024;107:104411. doi: 10.1016/j.etap.2024.104411 38452961

[42] Leslie HA, van Velzen MJM, Brandsma SH, Vethaak AD, Garcia-Vallejo JJ, Lamoree MH. Discovery and quantification of plastic particle pollution in human blood. Environ Int. 2022;163:107199. doi: 10.1016/j.envint.2022.107199 35367073

[43] Uogintė I, Pleskytė S, Pauraitė J, Lujanienė G. Seasonal variation and complex analysis of microplastic distribution in different WWTP treatment stages in Lithuania. Environ Monit Assess. 2022;194(11):829. doi: 10.1007/s10661-022-10478-x 36161366 PMC9511462

[44] Hajji S, Ben-Haddad M, Abelouah MR, De-la-Torre GE, Alla AA. Occurrence, characteristics, and removal of microplastics in wastewater treatment plants located on the Moroccan Atlantic: the case of Agadir metropolis. Sci Total Environ. 2023;862:160815. doi: 10.1016/j.scitotenv.2022.160815 36502989

[45] Patil S, Kamdi P, Chakraborty S, Das S, Bafana A, Krishnamurthi K, et al. Characterization and removal of microplastics in a sewage treatment plant from urban Nagpur, India. Environ Monit Assess. 2022;195(1):47. doi: 10.1007/s10661-022-10680-x 36309917

[46] Alavian Petroody SS, Hashemi SH. Wastewater treatment plants as a pathway for the release of microplastics into the environment: investigation of sludge and treated effluent of Sari wastewater treatment plant. Amirkabir Journal of Civil Engineering. 2021;53(9):3751–62. doi: 10.22060/CEEJ.2020.18097.6766

[47] Arregocés-Garcés R, Garcés-Ordóñez O, Vivas-Aguas L-J, Canals M. Microplastics transfer from a malfunctioning municipal wastewater oxidation pond into a marine protected area in the Colombian Caribbean. Regional Studies in Marine Science. 2024;69:103361. doi: 10.1016/j.rsma.2023.103361

[48] Tadsuwan K, Babel S. Unraveling microplastics removal in wastewater treatment plant: a comparative study of two wastewater treatment plants in Thailand. Chemosphere. 2022;307(Pt 2):135733. doi: 10.1016/j.chemosphere.2022.135733 35870611

[49] Hashempour Y, Zazouli MA, Jabari A. The plastisphere: microbial communities and their role in microplastic biodegradation in aquatic environments: a review. J-Mazand-Univ-Med-Sci. 2026;35(253):110.

[50] Parashar N, Hait S. Occurrence and removal of microplastics in a hybrid growth sewage treatment plant from Bihar, India: a preliminary study. Journal of Cleaner Production. 2022;376:134295. doi: 10.1016/j.jclepro.2022.134295

[51] Franco AA, Arellano JM, Albendín G, Rodríguez-Barroso R, Quiroga JM, Coello MD. Microplastic pollution in wastewater treatment plants in the city of Cádiz: Abundance, removal efficiency and presence in receiving water body. Science of The Total Environment. 2021;776:145795. doi: 10.1016/j.scitotenv.2021.145795

[52] Sakali A, Coello D, Brigui J, Albendín G, Arellano J, Quiroga JM, et al. Annual estimates of microplastics in municipal sludge treatment plants in southern Spain. Journal of Water Process Engineering. 2022;49:102956. doi: 10.1016/j.jwpe.2022.102956

[53] Asban P, Dehbandi R, Mohammadi MJ, Ghanbari S, Takdastan A, Jaafarzadeh Haghighifard N. Identification and distribution of microplastics in road dust and urban soils: a study in The Southwest of Iran. Environ Health Eng Manag. 2026;13:1309. doi: 10.34172/ehem.1309

[54] Campanale C, Massarelli C, Savino I, Locaputo V, Uricchio VF. A detailed review study on potential effects of microplastics and additives of concern on human health. Int J Environ Res Public Health. 2020;17(4):1212. doi: 10.3390/ijerph17041212 32069998 PMC7068600

[55] Van Do M, Le TXT, Vu ND, Dang TT. Distribution and occurrence of microplastics in wastewater treatment plants. Environmental Technology & Innovation. 2022;26:102286. doi: 10.1016/j.eti.2022.102286

[56] Asadi A, Khodadost F, Pirsaheb M, Davoodi R. Abundance, characteristics, fate, and removal of microplastics during municipal wastewater treatment plant in the west of Iran: the case of Kermanshah city. Environmental Monitoring and Assessment. 2023;195(7):857. doi: 10.1007/s10661-023-11473-6?urlappend=?utm_source=researchgate.net&utm_medium=article 37330424

[57] Wang W, Wang C. Polyurethane for biomedical applications: a review of recent developments. The Design and Manufacture of Medical Devices. Elsevier. 2012. p. 115–51. doi: 10.1533/9781908818188.115

[58] Bouattour Y, Wasiak M, Bernard L, Pinguet J, Richard D, Le Rouzo-Grèves M, et al. Quantification of bis(2-ethylhexyl) phthalate released by medical devices during respiratory assistance and estimation of patient exposure. Chemosphere. 2020;255:126978. doi: 10.1016/j.chemosphere.2020.126978 32417514

[59] Hecht ES, Yeh GK, Zhang K. Evidence of free radical generation from the interaction of polyethylene glycol with PVC medical tubing. J Pharm Biomed Anal. 2021;197:113955. doi: 10.1016/j.jpba.2021.113955 33607502

[60] Joseph B, James J, Kalarikkal N, Thomas S. Recycling of medical plastics. Advanced Industrial and Engineering Polymer Research. 2021;4(3):199–208. doi: 10.1016/j.aiepr.2021.06.003

[61] Paim AAM, de Andrade MC, Steffens F. The use of textile materials for respiratory protection in times of pandemics: a systematic review and analysis. RJTA. 2023;28(4):493–506. doi: 10.1108/rjta-04-2022-0041

[62] Fox S, Stefánsson H, Peternell M, Zlotskiy E, Ásbjörnsson EJ, Sturkell E, et al. Physical characteristics of microplastic particles and potential for global atmospheric transport: a meta-analysis. Environ Pollut. 2024;342:122938. doi: 10.1016/j.envpol.2023.122938 37981185

[63] Zhou G, Wang Q, Zhang J, Li Q, Wang Y, Wang M, et al. Distribution and characteristics of microplastics in urban waters of seven cities in the Tuojiang River basin, China. Environ Res. 2020;189:109893. doi: 10.1016/j.envres.2020.109893 32979998

[64] Dusaucy J, Gateuille D, Perrette Y, Naffrechoux E. Microplastic pollution of worldwide lakes. Environ Pollut. 2021;284:117075. doi: 10.1016/j.envpol.2021.117075 33894537

[65] Zhang J, Hong L, Wu K, Wang Q, Li M, Li X, et al., editors. Research progress of microplastic pollution status and risk assessment of typical Rivers in China. Journal of Physics: Conference Series; 2024: IOP Publishing. doi: 10.1088/1742-6596/2706/1/012087

[66] Simon-Sánchez L, Vianello A, Kirstein IV, Molazadeh M-S, Lorenz C, Vollertsen J. Assessment of microplastic pollution and polymer risk in the sediment compartment of the Limfjord, Denmark. Sci Total Environ. 2024;950:175017. doi: 10.1016/j.scitotenv.2024.175017 39059658

[67] Lamichhane G, Acharya A, Marahatha R, Modi B, Paudel R, Adhikari A, et al. Microplastics in environment: global concern, challenges, and controlling measures. Int J Environ Sci Technol (Tehran). 2023;20(4):4673–94. doi: 10.1007/s13762-022-04261-1 35638092 PMC9135010

[68] Zhang Y, Jiang H, Bian K, Wang H, Wang C. A critical review of control and removal strategies for microplastics from aquatic environments. Journal of Environmental Chemical Engineering. 2021;9(4):105463. doi: 10.1016/j.jece.2021.105463

[69] Lv X, Dong Q, Zuo Z, Liu Y, Huang X, Wu W-M. Microplastics in a municipal wastewater treatment plant: fate, dynamic distribution, removal efficiencies, and control strategies. Journal of Cleaner Production. 2019;225:579–86. doi: 10.1016/j.jclepro.2019.03.321

